# Effects of dexmedetomidine on perioperative neurocognitive disorders in elderly patients undergoing non-cardiac surgery: a scoping review

**DOI:** 10.3389/fnins.2026.1824272

**Published:** 2026-05-11

**Authors:** Jinxiang Xie, Bokang Yang, Jiayi Xie, Abdulrahman Khaled Alwesabi, Chengying Ji, Zhaohui Gao, Boxiong Gao, Qijing Liu, Yatao Liu

**Affiliations:** 1 The First School of Clinical Medicine, Lanzhou University, Lanzhou, Gansu, China; 2 Department of Anesthesiology and Surgery, First Hospital of Lanzhou University, Lanzhou, Gansu, China

**Keywords:** aged, delayed neurocognitive recovery, dexmedetomidine, non-cardiac surgery, perioperative neurocognitive disorders, postoperative delirium, postoperative neurocognitive disorder

## Abstract

**Background:**

Perioperative neurocognitive disorders (PNDs) are common and clinically significant complications in older surgical patients. Dexmedetomidine (DEX) has been investigated for neuroprotection; however, reported outcomes across PNDs subtypes remain inconsistent.

**Methods:**

Following PRISMA-ScR guidelines, we systematically searched PubMed, Embase, and Cochrane Library up to December 9, 2025. Eligible studies included patients aged ≥ 60 years undergoing non-cardiac surgery who received perioperative DEX. Data on study design, intervention characteristics, and cognitive outcomes were extracted and thematically synthesized.

**Results:**

Twenty-seven studies (*n* = 9,890 patients) were included. DEX effects varied by PNDs subtype and assessment timing. For delayed neurocognitive recovery (dNCR), 13 of 19 studies demonstrated improved cognitive performance or reduced incidence. For postoperative delirium (POD), results were heterogeneous: 6 trials reported significant incidence reduction, whereas 4 found no effect. None of the four studies assessing long-term postoperative neurocognitive disorder (pNCD) showed benefit. Proposed mechanisms include anti-inflammatory, antisympathetic, analgesic, and neuroprotective pathways. Substantial heterogeneity was observed in DEX protocols, cognitive tools, diagnostic criteria, and application of the updated nomenclature.

**Conclusion:**

DEX most consistently benefits dNCR, supporting its use in acute postoperative recovery. Its effect on POD is context-dependent, and no protective effect against long-term pNCD is evident. Considerable methodological diversity underscores the need for standardized diagnostic approaches and rigorously designed large-scale trials to clarify DEX’s role in perioperative cognitive protection.

## Introduction

Perioperative neurocognitive disorders (PNDs), particularly postoperative delirium (POD) and postoperative cognitive dysfunction (POCD), are prevalent and significant complications among older patients undergoing surgery (Wang et al., 2025). These disorders are strongly associated with adverse outcomes, including prolonged hospitalization, delayed functional recovery, increased mortality, and long-term cognitive decline (Dilmen et al., 2024; Deblois et al., 2025). Given the ongoing global demographic shift toward an aging population, the proportion of noncardiac surgical procedures performed on elderly individuals is steadily rising (GBD 2023 Cancer Collaborators, 2025; Hong and Smilowitz, 2025). Consequently, the prevention and standardized management of PNDs have become critically urgent priorities in clinical practice.

Dexmedetomidine (DEX), a highly selective *α*₂-adrenergic receptor agonist, has emerged as a commonly employed adjunct in the perioperative setting due to its favorable properties, including cooperative sedation, analgesia, anti-sympathetic effects, and minimal respiratory depression (Weerink et al., 2017; Ji et al., 2024; Li et al., 2025). In recent years, accumulating preclinical and clinical evidence has suggested that DEX may exert neuroprotective effects, potentially through mechanisms such as inhibiting inflammatory responses, reducing oxidative stress, and modulating neurotransmitter systems (Huang et al., 2024; Zheng et al., 2025). However, while numerous studies have explored the influence of DEX on postoperative neurocognitive function, the existing evidence remains fragmented, particularly regarding elderly patients undergoing noncardiac procedures, underscoring the need for a comprehensive synthesis of the available literature.

Despite a strong and converging rationale, studies examining the ability of DEX to prevent postoperative cognitive deficits have produced conflicting findings (Sun et al., 2021). This inconsistency likely arises from the multifactorial nature of PNDs. To better characterize these disorders, a new nomenclature was established in 2018, defining postoperative delirium (POD) as cognitive deficits occurring up to 1 week after surgery or before hospital discharge (whichever comes first), delayed neurocognitive recovery (dNCR) as cognitive deficits occurring up to 30 postoperative days, and postoperative neurocognitive disorder (pNCD) as cognitive deficits occurring from 30 days up to 1 year after surgery (Evered et al., 2018). Notably, previous reviews and meta-analyses that synthesized evidence on the cognitive effects of DEX have not systematically applied this updated framework. Evaluating the neuroprotective potential of DEX is further complicated by the fact that postoperative cognitive impairment encompasses multiple domains and follows various temporal trajectories (Brenna et al., 2025; Lahiri et al., 2025). Additionally, there is substantial heterogeneity across existing studies regarding DEX dosage, timing of administration, cognitive assessment tools, and follow-up duration, which challenges the integration of meaningful evidence (Huang et al., 2024; Lahiri et al., 2025; Zheng et al., 2025). Consequently, this study employs a scoping review methodology to systematically map the available evidence, clarify intervention effects, identify key sources of heterogeneity, and highlight directions for future research in this field.

## Methods

This scoping review adhered to the Preferred Reporting Items for Systematic Review and Meta-Analysis for Scoping Reviews (PRISMA-ScR) guidelines (Tricco et al., 2018). PubMed, Embase, and the Cochrane Library were systematically searched for relevant English-language articles published from the inception of these databases to December 9, 2025. The search strategy incorporated core keywords including “dexmedetomidine,” “geriatrics,” “aged,” “perioperative neurocognitive disorders,” “postoperative neurocognitive disorder,” “postoperative delirium,” “delayed neurocognitive recovery,” and “postoperative cognitive dysfunction,” along with their synonyms and Mesh/Emtree terms. In addition, the reference lists of included studies and relevant reviews were manually screened to identify additional publications. The complete search strategy is available in Supplementary material Search strategy.

This scoping review followed the PCC (Population, Concept, Context) framework (Peters et al., 2015). We included clinical trials and cohort studies involving patients aged ≥60 years undergoing noncardiac surgery with perioperative DEX administration (any dose, timing, or route). Eligible studies had to report outcomes related to PNDs subtypes, intervention effects, or assessment tools. Exclusions were cardiac surgery studies, participants under 60 years of age, non-original research (e.g., reviews, abstracts, animal studies), and studies without clear PNDs assessment outcomes.

Following deduplication in EndNote X9, two researchers independently screened records by title/abstract and subsequently by full-text against predefined eligibility criteria. Disagreements were resolved via team discussion or consultation with a third-party expert. The selection process is summarized in a PRISMA flow diagram. Relevant data regarding study design, patient and surgical characteristics, DEX regimen, cognitive assessments, and outcomes were extracted and analyzed.

Many studies investigating DEX and perioperative cognition predate the 2018 PNDs nomenclature update (Mahanna-Gabrielli et al., 2019). Given that these studies contain relevant data, their findings were integrated and reclassified into current cognitive subtypes based on assessment timing and clinical features (see Supplementary methods for details).

## Results

### Results of literature screening

**Figure 1:**
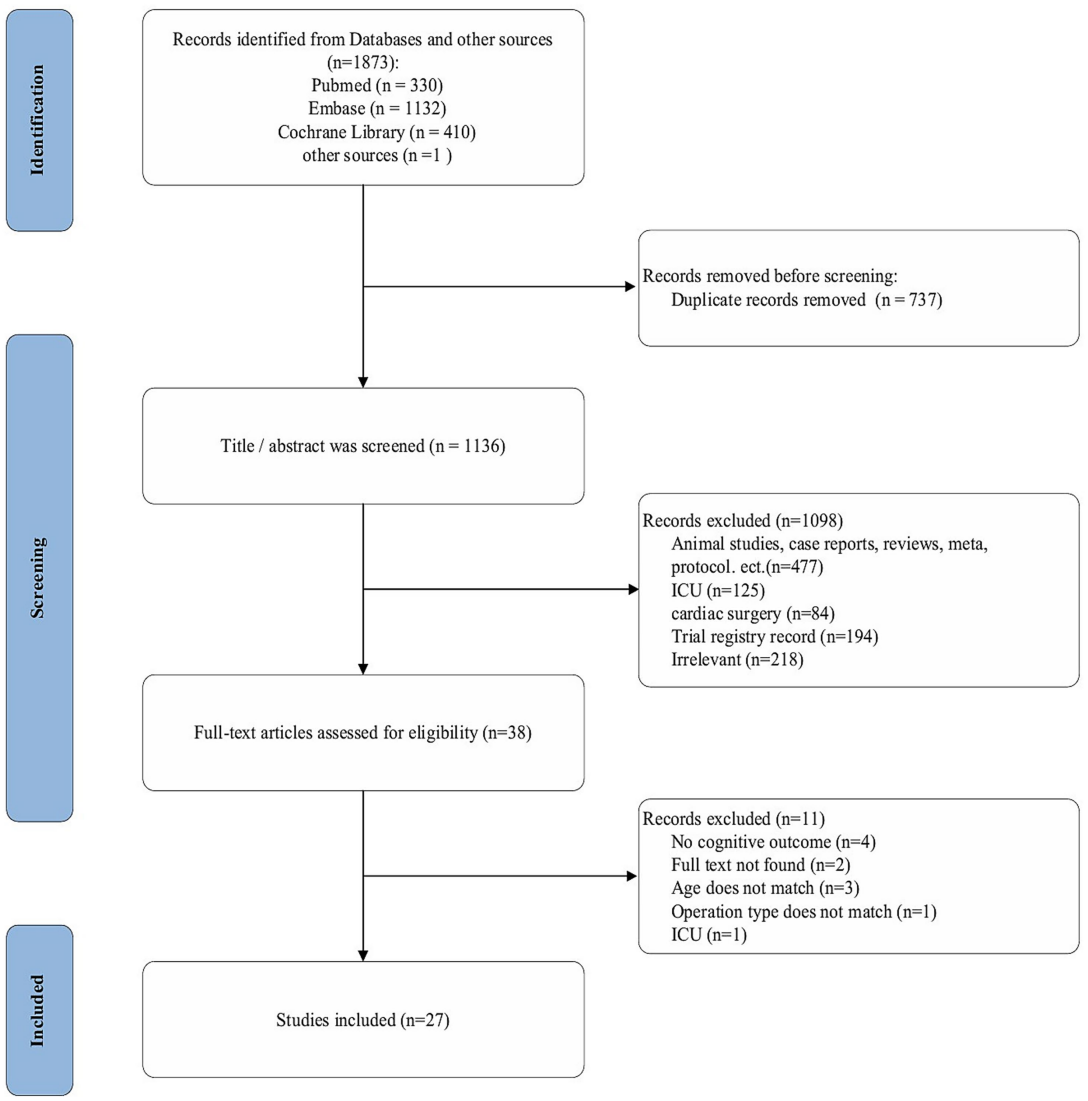
Flow chart of the study selection procedure.

A total of 1,873 relevant records were identified through preliminary retrieval, including 330 from PubMed, 1,132 from Embase, 410 from the Cochrane Library, and 1 from other sources. Following duplicate removal, 1,136 records were subjected to title/abstract screening. Of these, 1,098 records were excluded for the following main reasons: animal studies, case reports, reviews, meta-analyses, or study protocols (*n* = 477); studies involving ICU populations (*n* = 125) or cardiac surgery patients (*n* = 84); trial registrations (*n* = 194); and other literature irrelevant to the review objectives (*n* = 218). Full-text evaluation was conducted for the remaining 38 articles. Eleven articles were further excluded due to absence of neurocognitive outcomes (*n* = 4), unavailability of full text (*n* = 2), ineligible age range (*n* = 3), ineligible surgical type (*n* = 1), or ICU-based study population (*n* = 1). Ultimately, 27 studies were included in this scoping review. The detailed screening process is illustrated in Figure 1.

### Basic characteristics of included studies

This scoping review included 27 studies published between 2013 and 2025, with a marked increase in publication numbers in recent years (annual distribution shown in Supplementary Figure S1). The majority were conducted in China (*n* = 23, 85.2%), with one study each from Chile (Mehler et al., 2024), India (Kurup et al., 2023), Iran (Mansouri et al., 2019), and the United States (Deiner et al., 2017). All studies evaluated the effects of perioperative DEX on PNDs in older patients undergoing noncardiac surgery. Detailed characteristics of the included studies are presented in Supplementary Table S1.

Randomized controlled trials (RCTs) constituted 21 studies (77.8%), while six were cohort studies (22.2%, one prospective, five retrospective). Several RCTs adopted a multi-arm design comparing DEX with other agents [e.g., ketamine (Tao et al., 2024), remimazolam (Liao et al., 2023)] or different DEX dosages (Wei and Guo, 2023). Cohort studies were based on analyses of clinical records or prospective follow-up data.

The total sample size across all studies was 9,890 unique patients, with individual study sample sizes ranging from 23 to 5,591. By PNDs subtype, the numbers were: POD (11 studies, 7,750 patients), dNCR (19 studies, 2,658 patients), and pNCD (4 studies, 805 patients). The sum of these numbers exceeds 9,890 because several studies reported multiple outcomes. Most studies (*n* = 22, 81.5%) enrolled between 30 and 200 participants. The mean age (reported in 20 studies) ranged from 63.6 to 76.0 years, and the median age (reported in 7 studies) ranged from 64.0 to 82.0 years. The proportion of male patients varied substantially across studies (13.0 to 91.8%).

Regarding PNDs subtypes, 7 studies (25.9%) assessed only POD (Yin et al., 2020; Lu et al., 2021; Xin et al., 2021; Hu et al., 2022; Ye et al., 2024; Zhang et al., 2024; Hao et al., 2025), 13 (48.1%) only dNCR (Chen et al., 2015; Mansouri et al., 2019; Zhou et al., 2019; Li et al., 2020, 2024; Wang et al., 2020; Kurup et al., 2023; Liao et al., 2023; Bu et al., 2024; Huo et al., 2024; Mehler et al., 2024; Sun et al., 2024; Tao et al., 2024), 3 (11.1%) both POD and dNCR (Mei et al., 2018; Wei and Guo, 2023; Wu et al., 2023), 3 (11.1%) both dNCR and pNCD (Chen et al., 2013; Wang et al., 2019; Gao et al., 2022), and 1 (3.8%) both POD and pNCD (Deiner et al., 2017). Control interventions comprised normal saline placebo (40.7%), standard anesthesia (18.5%), other active drugs (29.6%), and different DEX doses (3.8%). Two studies (7.4%) compared DEX combined with ropivacaine nerve block to ropivacaine alone (Huo et al., 2024; Zhang et al., 2024).

Surgical types exhibited a distinct distribution. Twenty-one studies (77.8%) investigated a single specific procedure: the most common were elective total hip/knee arthroplasty and laparoscopic radical resection of gastrointestinal cancer, while other focused procedures included inguinal hernia repair (Zhang et al., 2024), endoscopic retrograde cholangiopancreatography (ERCP) (Sun et al., 2024), and open abdominal surgery (Kurup et al., 2023). Six studies (22.2%) used broader criteria (e.g., “major non-cardiac surgery”) (Chen et al., 2015; Deiner et al., 2017; Wang et al., 2019; Lu et al., 2021; Mehler et al., 2024; Hao et al., 2025). Overall, orthopedic and general surgeries represented the majority of included procedures (full percentage distribution shown in Supplementary Figure S2). General anesthesia was used in 18 studies (66.6%); 8 studies (29.6%) combined general anesthesia with regional nerve blocks, and 1 (3.8%) used combined spinal-epidural anesthesia (Wei and Guo, 2023).

**Figure 2:**
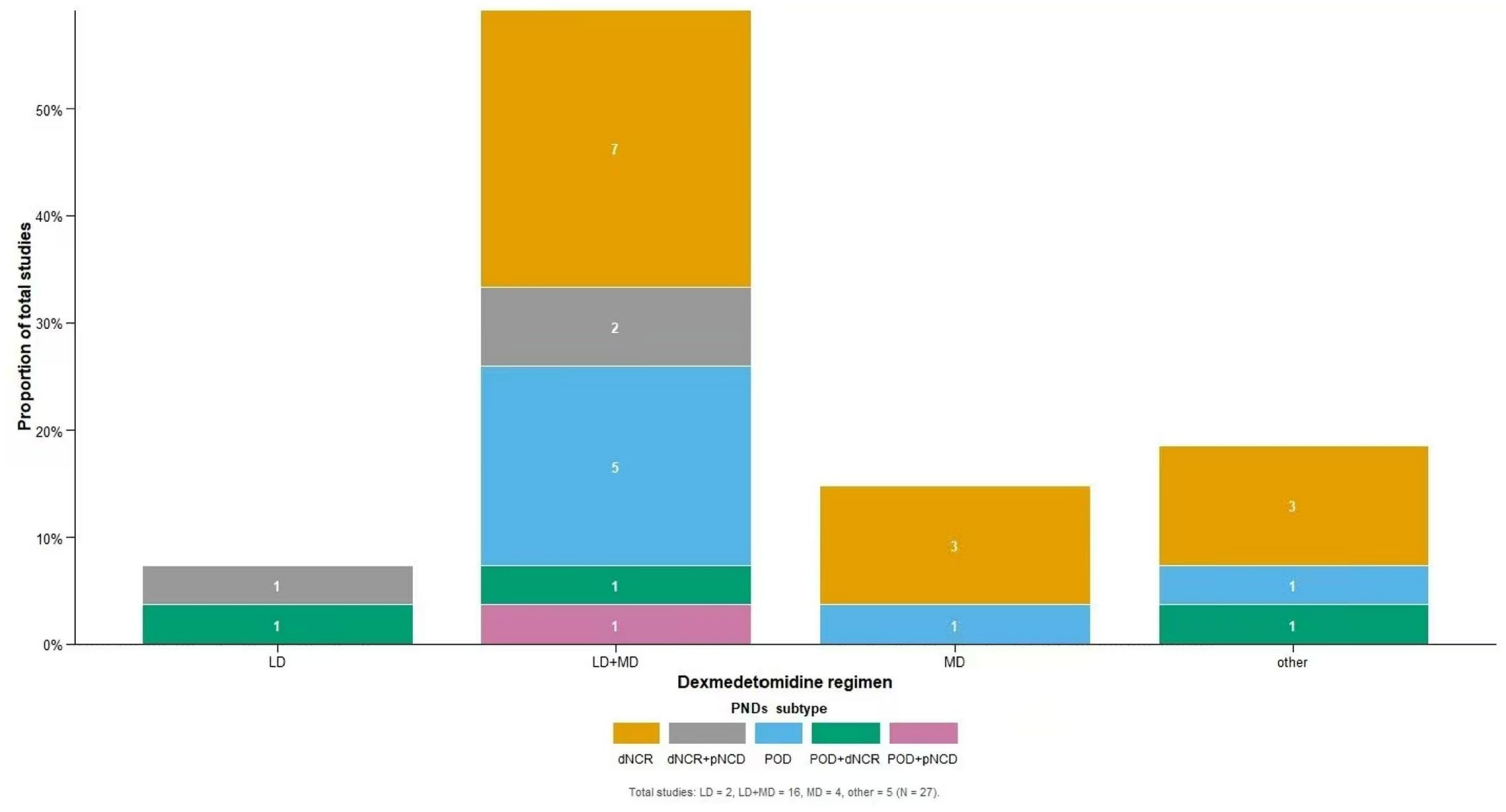
Distribution of dexmedetomidine administration regimens across included studies, stratified by PNDs subtype. The *x*-axis shows four categories of dexmedetomidine regimens: loading dose only (LD), loading dose plus maintenance dose (LD + MD), maintenance dose only (MD), and other routes (intranasal, tracheal mucosal, perineural, and patient-controlled analgesia). The *y*-axis represents the proportion of studies. Colors indicate different PNDs subtypes: delayed neurocognitive recovery (dNCR, orange), dNCR plus postoperative neurocognitive disorder (dNCR+pNCD, gray), postoperative delirium (POD, light blue), POD plus dNCR (green), and POD plus pNCD (purple). Numbers within each segment denote the count of studies. Total studies: LD = 2, LD + MD = 16, MD = 4, other = 5.

DEX administration was highly variable. Administration routes included single intravenous injection, loading dose (LD) only, maintenance dose (MD) only, LD + MD, intranasal, tracheal mucosal, perineural, and patient-controlled analgesia. The distribution of these regimens across the different PNDs subtypes is illustrated in Figure 2. Regarding specific dosing, loading doses ranged from 0.2 to 2.0 μg /kg (most commonly 0.5 μg/kg), and maintenance infusions ranged from 0.1 to 1.0 μg·kg^−1^·h^−1^ (most commonly 0.4 μg·kg^−1^·h^−1^). The dose used for single-shot nerve block was 1 μg/kg. One study evaluated three loading-dose gradients (0.2, 0.4, 0.6 μg/kg) (Wei and Guo, 2023), and another used 2 μg/kg for patient-controlled analgesia (Gao et al., 2022).

### Effects of DEX on POD

Eleven studies examined the effect of DEX on POD: seven exclusively, and four in conjunction with other outcomes. Five studies designated POD as the primary endpoint, and six as a secondary endpoint. The effects of DEX on POD are summarized in Table 1.

**Table 1:** Overview of studies reporting on postoperative delirium (POD) in elderly non-cardiac surgery patients.

Study	Baseline cognitive assessment	POD Assessment Tools	Assessment Time Points	POD diagnostic criteria	Endpoint priority	Incidence of POD (%)^a^	Effect of DEX
Hao et al. (2025)	Yes/MMSE	3D-CAM	2×/day (10:00 a.m., 4:00 p.m.), consecutive 7 days after surgery or until discharge	Positive at any follow-up	Secondary	8.3 vs. 11.5	Positive
Zhang et al. (2024)	No	CAM-CR	1 day after surgery	Score ≥22 = delirium	Secondary	2.1 vs. 17	Positive
Ye et al. (2024)	No	CAM	2×/day, the first 48 h after surgery	NR	Primary	11.7 vs. 14.8	No effect
Wu et al. (2023)	Yes/MMSE	3D-CAM	2×/day until discharge	NR	Secondary	5.5 vs. 12.7	No effect
Wei and Guo (2023)	Yes/MMSE	Delirium rating scale	NR	Total score ≥ 18 points or most severe items ≥15	Secondary	0 vs. 2.56 vs. 7.89	Dose-dependent effect
Xin et al. (2021)	Yes/MMSE; MoCA; CDR	3D-CAM	2×/day (8 to 10 a.m., 6 to 8 p.m.), consecutive 7 days after surgery	Acute onset/fluctuating + inattention + (disorganized thinking or altered consciousness)	Primary	10 vs. 33	Positive
Lu et al. (2021)	No	CAM	2×/day, consecutive 3 days after surgery	NR	Secondary	1 day after surgery: 11.9 vs. 13.0; 2 days after surgery: 7.3 vs. 5.7; 3 days after surgery: 5.2 vs. 3.6	No effect
Hu et al. (2022)	Yes/NR	CAM	2×/day (9 to 11 a.m., 3 to 5 p.m.), consecutive 4 days after surgery	NR	Primary	ITT: 16.7 vs. 36.8; PP: 19.5 vs. 42.7	Positive
Yin et al. (2020)	Yes/MMSE	NR	NR	Acute onset/fluctuating + inattention + (disorganized thinking or altered consciousness)	Secondary	6.45 vs. 41.37	Positive
Mei et al. (2018)	Yes/MMSE	CAM	1–3 days after surgery	NR	Primary	7 vs. 16	Positive
Deiner et al. (2017)	Yes/MMSE	Structured delirium assessment^b^	consecutive 5 days after surgery or until discharge	NR	Primary	12.2 vs. 11.4	No effect

3D-CAM, 3D-Confusion Assessment Method; CAM, Confusion Assessment Method; CAM-CR, Chinese version of the Confusion Assessment Method; CDR, Clinical Dementia Rating; DEX, Dexmedetomidine; ITT, intention-to-treat analysis; MMSE, Mini Mental Status Examination; MoCA, Montreal Cognitive Assessment; NR, not reported; POD, Postoperative delirium; PP, per-protocol analysis; TIVA, Total Intravenous Anesthesia.

a Data in “Incidence of POD (%)” are presented as intervention group vs control group. For detailed group allocation and comparison protocols, refer to Supplementary Table S1.

b Structured delirium assessment consisted of the Delirium Symptom Interview, abbreviated digit span, MMSE, CAM, and Memorial Delirium Assessment Scale.

Significant heterogeneity was observed in baseline cognitive screening tools, POD assessment methods, and diagnostic criteria. For baseline cognitive assessment, the Mini-Mental State Examination (MMSE) was used in six studies, one study employed a combination of multiple tools (Xin et al., 2021), and one did not specify the assessment instrument (Hu et al., 2022). POD was most frequently evaluated using the Confusion Assessment Method (CAM) or the Chinese version of the Confusion Assessment Method (CAM-CR); or the 3D-Confusion Assessment Method (3D-CAM), with other scales applied in the remaining studies. Assessment frequency was generally twice daily postoperatively, but assessment duration varied from 24 h to 7 days; only six studies fully reported detailed assessment protocols. Diagnostic criteria were explicitly reported in only five studies, most commonly based on CAM criteria.

In control groups, POD incidence ranged from 3.6% (Lu et al., 2021) to 42.7% (Hu et al., 2022), compared with 0% (Wei and Guo, 2023) to 19.5% (Hu et al., 2022) in DEX-treated groups. Six studies reported a statistically significant reduction in POD incidence following DEX administration. For example, Yin et al. (2020) observed a decrease from 41.37% in the saline control group to 6.45% in the DEX group (Yin et al., 2020). Four studies found no significant effect, and one study reported a non-significant dose-dependent trend.

Proposed mechanisms underlying the protective effect of DEX against POD include its antisympathetic and sedative properties, analgesic and opioid-sparing effects, anti-inflammatory activity, neuroprotection with blood–brain barrier stabilization, hemodynamic modulation, and improvement in postoperative sleep quality.

### Effects of DEX on dNCR

Nineteen studies were included to evaluate the impact of DEX on dNCR. Of these, 13 focused exclusively on dNCR, while 6 reported composite cognitive and clinical outcomes; 14 studies designated dNCR as the primary endpoint. The effects of DEX on dNCR are presented in Table 2.

**Table 2:** Overview of studies reporting on delayed neurocognitive recovery (dNCR) in elderly non-cardiac surgery patients.

Study (year)	Baseline cognitive assessment	dNCR assessment tools	Assessment time points	dNCR diagnostic criteria	Endpoint priority	Incidence of dNCR (%)^a^	Effect of DEX
Tao et al. (2024)	Yes/MMSE; MoCA	MMSE; MoCA	1 day after surgery	Decrease ≥1 SD from baseline	Primary	24.5 vs. 11.3 vs. 32.1	Positive
			3 days after surgery			15.1 vs. 9.4 vs. 18.9	
Sun et al. (2024)	Yes/MMSE	MMSE	1 day after surgery	|Z| ≥ 1.96	Primary	10 vs. 30	Positive
			3 days after surgery			2.5 vs. 17.5	
			5 days after surgery			0 vs. 0	
Mehler et al. (2024)	Yes/MoCA	MoCA	1 hour after RoR	NR	Primary	NR	No effect
Li et al. (2024)	Yes/MoCA	MoCA	12 h after surgery	NR	Primary	NR	Positive
			24 h after surgery				
			72 h after surgery				
			7 days after surgery				
Huo et al. (2024)	Yes/MMSE	MMSE	1 day after surgery	Decrease ≥2 points from baseline	Primary	14.73 vs. 5.08 vs. 18.67	Positive
			7 days after surgery			9.30 vs. 1.69 vs. 12.00	
Bu et al. (2024)	Yes/MMSE	MMSE	1 day after surgery	Score < 27 points	Primary	11.86 vs. 27.59	Positive
			3 days after surgery			11.86 vs. 24.14	
			5 days after surgery			5.08 vs. 18.97	
			7 days after surgery			1.69 vs. 5.17	
Wu et al. (2023)	Yes/MMSE	MMSE	At discharge	NR	Secondary	NR	No effect
Wei and Guo (2023)	Yes/MMSE	MMSE	1 day after surgery	NR	Secondary	NR	Dose-dependent effect
			3 days after surgery				
Liao et al. (2023)	Yes/MMSE; MoCA	MMSE; MoCA	1 day after surgery	Decrease ≥1 SD from baseline	Primary	17.1 vs. 40 vs. 17.6	Positive
			3 days after surgery			8.6 vs. 28.6 vs. 8.8	
			7 days after surgery			NR	
Kurup et al. (2023)	Yes/MMSE; MoCA	MMSE; MoCA; Stroop test; Porteus Maze test; Trail making test	3 days after surgery	>20% reduction in any 2 tests	Primary	24.1 vs. 29.03	No effect
Gao et al. (2022)	Yes/MoCA-B	MoCA-B	1 day after surgery	NR	Secondary	NR	Positive
			2 days after surgery				
			At discharge				
Wang et al. (2020)	Yes/MMSE	MMSE	1 day after surgery	Decrease ≥2 points from baseline	Primary	20 vs. 40	Positive
Li et al. (2020)	Yes/MMSE	MMSE	After the recovery from anesthesia	NR	Secondary	NR	No effect
Bao et al. (2020)	Yes/MMSE	MMSE	1 day after surgery	NR	Primary	8.42 vs. 22.89	Positive
			3 days after surgery				
Wang et al. (2019)	Yes/MMSE	MMSE	5–7 days after surgery	Decrease ≥1 SD from baseline	Primary	24.5 vs. 28.0	No effect
Mansouri et al. (2019)	Yes/MMSE	MMSE	24 h after surgery	Score < 20 points	Primary	12 vs. 14 vs. 24	Positive
			1 week after surgery			12 vs. 8 vs. 20	
Mei et al. (2018)	Yes/MMSE	MMSE	3 days after surgery	NR	Secondary	NR	Positive
			7 days after surgery				
Chen et al. (2015)	Yes/MMSE	MMSE	1 day after surgery	Score < 24 points	Primary	9.20 vs. 21.31	Positive
Chen et al. (2013)	Yes/MMSE	MMSE	1 week after surgery	NR	Primary	NR	Positive

dNCR,delayed neurocognitive recovery; DEX,dexmedetomidine; MMSE, Mini Mental State Examination; MoCA, Montreal Cognitive Assessment; MoCA-B, Montreal Cognitive Assessment-Basic; NR, not reported; PENGB,pericapsular nerve group block; RoR,recovery of response; SD, standard deviation.

a Data in “Incidence of dNCR (%)” are presented as intervention group vs control group. For detailed group allocation and comparison protocols, refer to Supplementary Table S1.

Substantial heterogeneity was noted across the included studies with regard to baseline and postoperative cognitive assessment methodologies, as well as diagnostic criteria for dNCR. All studies performed preoperative cognitive screening, with MMSE or Montreal Cognitive Assessment (MoCA) serving as the dominant tools, used either alone (16 studies) or in combination (3 studies). These same assessment scales were also primarily adopted for postoperative dNCR evaluation in most trials; one study utilized the MoCA-Basic scale (Gao et al., 2022), and another supplemented subjective cognitive scales with objective neuropsychological tests (e.g., Stroop test, Porteus Maze test) (Kurup et al., 2023). Postoperative cognitive assessments were performed at diverse time points ranging from immediate emergence from anesthesia to 7 days postoperatively, with postoperative days 1 and 3 being the most frequently adopted assessment timepoints. Diagnostic criteria for dNCR varied markedly across studies, including a postoperative cognitive score reduction of ≥1 standard deviation from baseline (Tao et al., 2024), a score drop of ≥2 points (Huo et al., 2024), a score falling below a predefined cutoff value (Bu et al., 2024), or other customized statistical thresholds (Sun et al., 2024); notably, 9 studies did not explicitly report detailed diagnostic criteria for dNCR.

The majority of included studies documented significantly higher cognitive scale scores in the DEX group relative to control groups at multiple postoperative assessment intervals. For instance, Sun et al. (2024) reported higher MMSE scores in the DEX group compared with the saline control group on postoperative days 1, 3, and 5 (Sun et al., 2024). The incidence of dNCR ranged from 0% (Sun et al., 2024) to 40.0% (Wang et al., 2020) in control groups, versus 0% (Sun et al., 2024) to 24.5% (Tao et al., 2024) in DEX-treated groups. Thirteen studies verified that DEX administration significantly lowered the incidence of dNCR. By contrast, 5 studies detected no significant intergroup difference in dNCR incidence. A single study by Wei and Guo (2023) indicated a dose-dependent association, with lower-dose DEX cohorts exhibiting better postoperative cognitive scores than higher-dose DEX cohorts.

Proposed mechanisms underlying the beneficial effects of DEX against dNCR include its anti-inflammatory, antisympathetic, sedative, and neuroprotective properties, as well as its analgesic effects and opioid-sparing potential.

### Effects of DEX on pNCD

Four studies examining combined outcomes assessed the impact of DEX on pNCD, all of which treated pNCD as a secondary endpoint (Chen et al., 2013; Deiner et al., 2017; Wang et al., 2019; Gao et al., 2022). The effects of DEX on pNCD is presented in Table 3.

**Table 3:** Overview of studies reporting on postoperative neurocognitive disorder (pNCD) in elderly non-cardiac surgery patients.

Study (year)	Baseline cognitive assessment	pNCD assessment tools	Assessment time points	pNCD diagnostic criteria	Endpoint priority	Incidence of pNCD (%)^a^	Effect of DEX
Gao et al. (2022)	Yes/MoCA-B	MoCA-B	1 month after surgery	NR	Secondary	NR	No effect
			1 year after surgery				
Wang et al. (2019)	Yes/MMSE	MMSE	3 months after surgery	Postoperative MMSE score decreased by ≥1 SD from baseline	Secondary	12.9 vs. 11.3	No effect
Deiner et al. (2017)	Yes/MMSE	MMSE	3 months after surgery	NR	Secondary	NR	No effect
			6 months after surgery				
Chen et al. (2013)	Yes/MMSE	MMSE	1 month after surgery	NR	Secondary	NR	No effect

DEX,dexmedetomidine; MMSE, Mini Mental State Examination; MoCA-B, Montreal Cognitive Assessment-Basic; NR, not reported; pNCD,postoperative neurocognitive disorder; SD, standard deviation.

a Data in “Incidence of pNCD (%)” are presented as intervention group vs control group. For detailed group allocation and comparison protocols, refer to Supplementary Table S1.

All studies used the MMSE for preoperative baseline assessment. Postoperative cognitive evaluation was performed at various time points (1, 3, 6, and 12 months), primarily using the MMSE and MoCA-B. Only one study explicitly defined pNCD as a postoperative MMSE score decline of ≥1 standard deviation from baseline; the remaining three did not specify diagnostic criteria.

None of the four studies found a significant effect of DEX on pNCD incidence. Given the limited number of included studies and heterogeneity in assessment timing, the effect of DEX on long-term pNCD in elderly non-cardiac surgery patients remains uncertain.

## Discussion

Adhering to the PRISMA-ScR reporting guidelines, this scoping review systematically synthesized evidence from 27 studies (*n* = 9,890) on DEX and PNDs in elderly patients undergoing non-cardiac surgery, using the updated PNDs nomenclature. Our synthesis provides a comprehensive evidence map across cognitive subtypes. The findings indicate that the benefits of DEX appear to be time-dependent, with the most consistent effects observed for short-term cognitive impairment—particularly in reducing dNCR within 7 days postoperatively. The effect on acute disturbances such as POD is modest but accompanied by significant heterogeneity across studies. In contrast, DEX showed no significant impact on long-term pNCD. This pattern suggests that the neuroprotective potential of DEX may depend on both the timing of intervention and the severity of neural injury, with optimal efficacy likely achieved when administered early during active neuroinflammation, while pathophysiological processes remain potentially reversible (Paeschke et al., 2017; Wang et al., 2022).

This review identified significant heterogeneity in the reported effects of DEX on POD among elderly non-cardiac surgery patients, stemming primarily from three aspects. First, patient selection bias likely played a role. Studies reporting positive outcomes often enrolled higher-risk populations (e.g., hip fracture or major thoracic surgery), in which greater surgical trauma and inflammation may provide a wider therapeutic window for the anti-inflammatory effects of DEX (Yin et al., 2020; Hu et al., 2022). In contrast, studies with negative results frequently included mixed surgical cohorts, in which patient heterogeneity could dilute any benefit observed in specific subgroups (Deiner et al., 2017; Lu et al., 2021). Second, variability in assessment methods influenced outcome detection and results. Studies using the structured 3D-CAM instrument, which improves detection of subsyndromal delirium, were more likely to report a protective effect of DEX, whereas those using the traditional CAM or less frequent assessments yielded inconsistent conclusions (Marcantonio et al., 2014). Third, a potential dose–response paradox was observed in this review. For enhanced clarity and reproducibility, we herein define low-dose DEX as a LD ≤ 0.5 μg/kg and/or MD ≤ 0.4 μg·kg^−1^·h^−1^, and high-dose DEX as a LD > 0.5 μg/kg and/or MD > 0.4 μg·kg^−1^·h^−1^, consistent with widely adopted stratifications in perioperative neurocognitive research (Wei and Guo, 2023; Zeng et al., 2019; Li et al., 2021). Notably, a recent large-scale retrospective cohort study further validated this dose-effect pattern by demonstrating that the optimal cumulative dose for preventing postoperative delirium ranged from 0.25 to 0.35 μg·kg^−1^, with doses exceeding 0.40 μg·kg^−1^ losing protective efficacy (Ahrens et al., 2026). High-dose regimens may increase POD risk, possibly due to excessive sedation and reduced cerebral perfusion, and have been associated with lower early postoperative MMSE scores (Gautam et al., 2020; Wei and Guo, 2023). Conversely, improvement in dNCR may require reaching an anti-inflammatory dose threshold (Kim et al., 2017). Importantly, a protective effect against POD is most consistently observed with low-to-moderate LD (approximately 0.2–0.5 μg/kg) followed by low-rate MD, which achieve optimal sympatholysis, sedation, and anti-inflammation without over-sedation or hemodynamic compromise (Yin et al., 2020; Hu et al., 2022; Ahrens et al., 2026). These findings highlight the need for future standardized research to identify the optimal patient population and dosing window for DEX, and to clarify the mechanistic distinctions between POD and dNCR in order to guide precise perioperative cognitive protection strategies.

In studies examining dNCR, the effects of DEX demonstrated greater consistency. Among the 19 studies included in this review, 68.4% reported that DEX improved cognitive scores and reduced the incidence of dNCR within 1–7 days postoperatively. This consistency may be attributed to the more standardized assessment of dNCR and the closer alignment of DEX’s mechanisms with its underlying pathophysiology. The remaining five studies showed no significant effects, with heterogeneity likely arising from differences in dosing regimens, concomitant medications, and patient baseline characteristics. Notably, Wei and Guo (2023) confirmed a dose-dependent cognitive protective effect of DEX. The core mechanisms by which DEX improves dNCR include anti-inflammatory and neuroprotective actions, anti-sympathetic effects, and synergistic multi-drug interactions (Wang et al., 2025). DEX inhibits the release of cytokines such as IL-6 and TNF-*α* by activating central α₂-adrenergic receptors and the cholinergic anti-inflammatory pathway, while also modulating NF-κB signaling (Ma et al., 2020; Sun et al., 2024). Evidence further indicates that DEX reduces plasma S100-*β* protein levels, suppresses astrocyte activation, maintains regional cerebral oxygen saturation, and stabilizes hemodynamics via its sedative and analgesic properties—thereby lowering cortisol levels and mitigating stress-related cognitive impairment (Rao et al., 2024). Moreover, administration of DEX in combination with agents such as esketamine or ulinastatin has been shown to confer synergistic benefits. In summary, DEX exerts beneficial effects on dNCR through multiple targets and pathways, and combination therapy represents a promising clinical strategy. However, substantial heterogeneity in dosing regimens persists across studies. Future high-quality research is warranted to optimize treatment strategies and strengthen the evidence base.

The four studies included in this review examining the effect of DEX on pNCD consistently reported no significant benefit. This finding is clinically relevant, as pNCD involves persistent cognitive decline driven by chronic degenerative processes—such as neuronal loss and protein pathology—that are unlikely to be reversed by short-term perioperative intervention (Tasbihgou and Absalom, 2021; Oikonomou et al., 2026). Methodological limitations likely contributed to these negative results, including high attrition rates, confounding factors, limited sensitivity of cognitive screening tools, and a mismatch between brief DEX exposure and the prolonged clinical course of pNCD. Furthermore, all available studies treated pNCD as a secondary endpoint, with small sample sizes and often inadequate follow-up, limiting the ability to detect potential subtle effects. Because pNCD is influenced by long-term factors such as baseline cognitive reserve and genetic susceptibility, isolated pharmacological intervention offers limited utility (Evered and Silbert, 2018). A comprehensive perioperative brain health strategy—incorporating prehabilitation, intraoperative monitoring, and long-term postoperative follow-up—is therefore warranted (Cong et al., 2025; Gao et al., 2026). Within such a framework, DEX may play a supportive role, but its value for long-term cognitive protection requires validation in larger, long-term, high-quality studies.

This review identified significant heterogeneity across studies investigating the effects of DEX on PNDs in elderly non-cardiac surgery patients, arising from four main sources: intervention protocols, assessment methods, study populations, and conceptual frameworks. Variability in intervention protocols was most evident, with wide ranges in dosing, administration routes, and concomitant medications (Hao et al., 2025). Assessment methods also lacked standardization, differing in diagnostic tools, criteria, and follow-up timing across PNDs subtypes. Furthermore, study populations varied substantially; current evidence is predominantly derived from Chinese cohorts and shows considerable diversity in patient and surgical characteristics, limiting external generalizability. The evolution of PNDs nomenclature since 2018 has additionally introduced conceptual discrepancies between earlier and later studies (Lahiri et al., 2025). These interconnected sources of heterogeneity contribute to the inconsistent effects reported for DEX.

This review has several limitations. First, the evidence is geographically concentrated, with 85.2% of studies originating from China. This predominance likely reflects the widespread clinical use of DEX in China, the large elderly surgical population, and active local research interest. Therefore, the findings may have limited generalizability to other populations, and international validation is needed. Second, most studies had small sample sizes below 200 and consisted primarily of RCTs, lacking support from large-scale real-world data. Third, substantial heterogeneity in DEX dosing, assessment methods, and follow-up timing precluded quantitative synthesis. Fourth, long-term cognitive outcomes were rarely reported. Finally, exclusion of grey literature may have introduced publication bias (Keith et al., 2025).

Based on the synthesis of current evidence, future research should progress from methodological standardization toward precision medicine in clinical practice. Establishing a standardized assessment framework for PNDs subtypes is fundamental to ensuring comparability across studies (Lahiri et al., 2025). Conducting large-scale, multicenter trials will be crucial to validate the effects of DEX and define optimal dosing regimens (Zeng et al., 2019). Integrating multimodal tools is needed to clarify the underlying mechanisms (Wang et al., 2025). Developing predictive models can enable personalized treatment strategies (Wei et al., 2026). Finally, enhancing global representativeness through internationally diverse studies is essential for generating generalizable evidence (Brenna et al., 2025).

## Conclusion

In summary, this review indicates that the effects of dexmedetomidine on perioperative neurocognitive disorders are both subtype-specific and time-dependent. The most consistent benefit is observed for short-term cognitive recovery, supporting its proposed anti-inflammatory and neuroprotective properties during the acute postoperative period. For postoperative delirium, however, evidence remains inconsistent and appears dependent on specific patient characteristics and intervention protocols. Furthermore, current data do not demonstrate a clear protective effect against long-term cognitive decline. Substantial heterogeneity across studies underscores the importance of standardizing assessment methods and intervention designs. Moving forward, research should prioritize the development of unified diagnostic criteria and the conduct of large-scale, rigorously designed trials to optimize the targeted use of DEX in perioperative cognitive protection strategies.

## Data Availability

Publicly available datasets were analyzed in this study. This data can be found at “Data are from the articles cited in the reference list. No new datasets were generated.”
