# Supplementary material for: Effects of dexmedetomidine on perioperative neurocognitive disorders in elderly patients undergoing non-cardiac surgery: a scoping review
Source: Front Neurosci. 2026 May 11;20:1824272. doi: 10.3389/fnins.2026.1824272 (PMC13199336; doi:10.3389/fnins.2026.1824272)
Supplement: Supplementary file 1 [file Data_Sheet_1.docx]

Supplementary Material

# Search strategy : (inception – December 9, 2025)

| **#** | **Searches in PubMed** | **Results** |
| --- | --- | --- |
| 1 | "Dexmedetomidine"[Mesh] OR "dexmedetomidine"[Title/Abstract] OR "dexmedetomidine"[Keyword] OR "precedex"[Title/Abstract] OR "mpv 1440"[Title/Abstract] OR "dexmedetomidine hydrochloride"[Title/Abstract] | 11277 |
| 2 | "Geriatrics"[Mesh] OR "Aged"[Mesh] OR "Gerontology"[Title/Abstract] OR "Elderly"[Title/Abstract] OR "elderly patients"[Title/Abstract] | 3,913,272 |
| 3 | "Neurocognitive Disorders"[MeSH] OR "Postoperative Cognitive Complications"[MeSH] OR "Emergence Delirium"[MeSH] OR "perioperative neurocognitive disorders"[Title/Abstract] OR "perioperative neurocognitive dysfunction"[Title/Abstract] OR "PND"[Title/Abstract] OR “postoperative neurocognitive disorder” [Title/Abstract] OR "pNCD"[Title/Abstract] OR "postoperative cognitive dysfunction"[Title/Abstract] OR "POCD"[Title/Abstract] OR "postoperative delirium"[Title/Abstract] OR "POD"[Title/Abstract] OR "delayed neurocognitive recovery"[Title/Abstract] OR "dNCR"[Title/Abstract] | 381,554 |
| 4 | 1 AND 2 AND 3 | 330 |

| **#** | **Searches in Embase** | **Results** |
| --- | --- | --- |
| 1 | ‘Dexmedetomidine'/exp OR ‘dexmedetomidine’:ti,ab,kw OR ‘precedex’:ti,ab,kw OR ‘mpv 1440’:ti,ab,kw OR ‘dexmedetomidine hydrochloride’:ti,ab,kw | 28252 |
| 2 | ‘Geriatrics’/exp OR ‘Aged'/exp OR ‘Gerontology’:ti,ab,kw OR ‘Elderly’:ti,ab,kw OR ‘elderly patients’:ti,ab,kw | 4,707,702 |
| 3 | 'disorders of higher cerebral function'/exp OR 'postoperative cognitive dysfunction'/exp OR 'delirium'/exp OR ‘perioperative neurocognitive disorders’:ti,ab,kw OR ‘perioperative neurocognitive dysfunction’:ti,ab,kw OR ‘PND’:ti,ab,kw OR ‘postoperative neurocognitive disorder’ :ti,ab,kw OR ‘pNCD’:ti,ab,kw OR ‘POCD’:ti,ab,kw OR ‘postoperative cognitive dysfunction’:ti,ab,kw OR ‘postoperative delirium’:ti,ab,kw OR ‘POD’:ti,ab,kw OR ‘delayed neurocognitive recovery’:ti,ab,kw OR ‘dNCR’:ti,ab,kw | 1,253,034 |
| 4 | 1 AND 2 AND 3 | 1,132 |

| **#** | **Searches in the Cochrane Library** | **Results** |
| --- | --- | --- |
| 1 | MeSH descriptor: [Dexmedetomidine] explode all trees | 3279 |
| 2 | (dexmedetomidine):ti,ab,kw OR (precedex):ti,ab,kw OR (mpv 1440):ti,ab,kw OR (dexmedetomidine hydrochloride):ti,ab,kw | 12196 |
| 3 | 1 OR 2 | 12196 |
| 4 | MeSH descriptor: [Geriatrics] explode all trees | 297 |
| 5 | MeSH descriptor: [Aged] explode all trees | 287712 |
| 6 | (Gerontology):ti,ab,kw OR (Elderly):ti,ab,kw OR (elderly patients):ti,ab,kw | 65659 |
| 7 | 4 or 5 or 6 | 333067 |
| 8 | MeSH descriptor: [Neurocognitive Disorders] explode all trees | 19639 |
| 9 | MeSH descriptor: [Postoperative Cognitive Complications] explode all trees | 164 |
| 10 | MeSH descriptor: [Emergence Delirium] explode all trees | 530 |
| 11 | (perioperative neurocognitive disorders):ti,ab,kw OR (perioperative neurocognitive dysfunction):ti,ab,kw OR (PND):ti,ab,kw OR (postoperative neurocognitive disorder) :ti,ab,kw OR (pNCD):ti,ab,kw OR (POCD):ti,ab,kw OR (postoperative cognitive dysfunction):ti,ab,kw OR (postoperative delirium):ti,ab,kw OR (POD):ti,ab,kw OR (delayed neurocognitive recovery):ti,ab,kw OR (dNCR):ti,ab,kw | 7927 |
| 12 | 8 OR 9 OR 10 OR 11 | 26159 |
| 13 | 3 AND 7 AND 12 | 410 |

Supplementary Methods

The methodological approach for this scoping review was designed to systematically map and synthesize the available evidence on dexmedetomidine (DEX) and perioperative neurocognitive disorders (PNDs) in elderly patients undergoing non-cardiac surgery. The decision to employ a scoping review methodology stemmed from preliminary searches that revealed a rapidly expanding yet methodologically heterogeneous body of literature. This approach is particularly suited for clarifying key concepts, identifying evidence gaps, and characterizing the nature and sources of variation across studies, especially in fields where research practices are still evolving.

Data extraction was conducted using a standardized template to ensure consistency. Information collected from each included study covered several dimensions: general study characteristics (e.g., author, year, country, design, sample size); details of the patient population and surgical procedure (e.g., mean age, sex, surgery type, anesthetic technique); the specific DEX intervention protocol (e.g., dose, route, timing, duration); and comprehensive data on cognitive outcomes (e.g., PND subtype assessed, specific assessment tools and diagnostic criteria used, timing of evaluations, and primary results such as incidence rates or score changes).

A critical challenge in synthesizing this literature is the historical evolution of terminology surrounding postoperative cognitive changes. Prior to the 2018 international consensus, a wide array of impairments was often grouped under the broad label of "postoperative cognitive dysfunction (POCD)." The contemporary framework distinguishes postoperative delirium (POD), delayed neurocognitive recovery (dNCR), and postoperative neurocognitive disorder (pNCD) as separate entities with distinct clinical features and time courses (Evered et al., 2018). To integrate findings from studies using older or inconsistent terminology, we implemented a post-hoc reclassification. This process was based on the timing and nature of the cognitive assessments described in each study. Assessments targeting acute disturbances in attention and awareness (e.g., using the CAM or 3D-CAM) within the first postoperative week were classified under POD. Evaluations of domain-specific cognitive performance (e.g., via MMSE or MoCA) conducted between emergence from anesthesia and 30 days post-surgery were categorized as dNCR. Cognitive testing performed from 30 days to 12 months postoperatively was considered indicative of pNCD. This reclassification allowed for a coherent synthesis of evidence across the temporal spectrum of PNDs within the modern conceptual framework.

# Supplementary Figures and Table

## Supplementary Figures


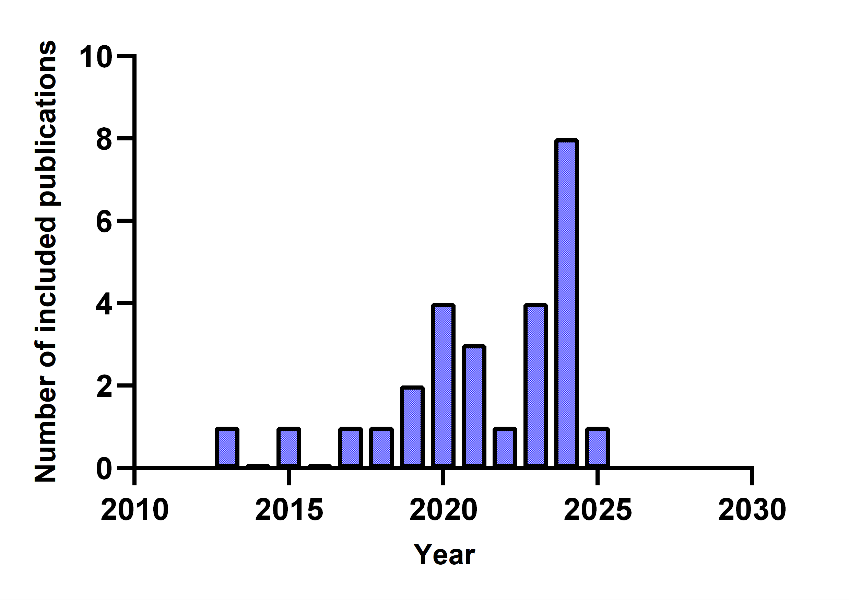


Figure S 1 Publications by year. Number of included publications arranged by year of publication. Literature was searched up to 2025-Dec-9; hence, the final bar represents an incomplete sample.


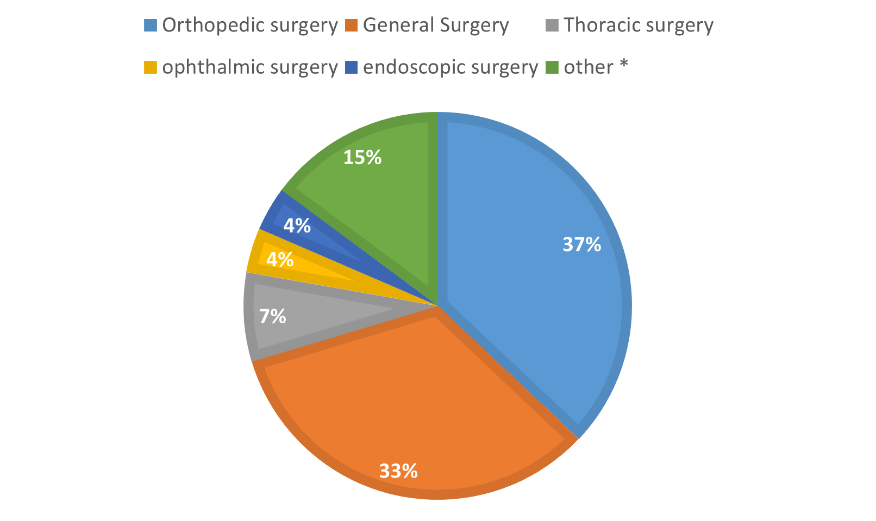


Figure S 2 Distribution of Surgical Procedures. *: Urology, Gynecology, Cataract surgery, etc.

**Supplementary Table**

Supplementary Table 1 Characteristics of 27 Included Studies in This Scoping Review.

| **Study (year)** | **Country** | **Study Design** | **PND Type(s)** | **Comparison** | **Age, y** | **Sex (% Male)** | **Sample size** | **Type of surgery** | **Anesthetic method** | **Dosage of DEX** |
| --- | --- | --- | --- | --- | --- | --- | --- | --- | --- | --- |
| Hao et al., 2025 | China | Prospective Cohort | POD | DEX vs no DEX | 69.0[66.0,74.0] vs 70.0[67.0,74.0] | 66.5% vs 56.0% | 5591(1148 vs 4443) | Non-cardiac surgery (otolaryngology, urology, gynecology, gastroenterology, orthopedics, thoracic surgery, hepatobiliary surgery) | GA | 0.2-1.0 μg・kg⁻¹・h⁻¹ MD |
| Zhang et al., 2024 | China | RCT | POD | DEX + ropivacaine vs ropivacaine | 74.7±6.1 vs 73.9±6.7 | 91.8% vs 85.7% | 98(49 vs 49) | Elective unilateral inguinal hernia repair | Transversus abdominis plane block (TAP block) + GA | 1 μg/kg BA |
| Ye et al., 2024 | China | RCT | POD | DEX vs no DEX | 73.0[66.0,82.0] vs 71.0[66.0,80.0] | 40.0% vs 42.6% | 121(60 vs 61) | Hip surgery | GA + Fascia iliaca block (FIB) | 0.7 μg/kg LD then 0.1 μg・kg⁻¹・h⁻¹ MD |
| Tao et al., 2024 | China | RCT | dNCR | DEX vs DEX + esketamine vs esketamine | 72.2±6.2 vs 71.9±7.7 vs 71.6±7.2 | 37.7% vs 37.7% vs 39.6% | 159 (53 vs 53 vs 53) | Elective lumbar spine surgery | GA | 0.4 µg/kg IV then 0.6 µg/kg Tracheal mucosal application |
| Sun et al., 2024 | China | RCT | dNCR | DEX vs saline | 68.2±2.2 vs 68.0±1.9 | 47.5% vs 50.0% | 80 (40 vs 40） | Elective ERCP | GA | 0.4 μg・kg⁻¹・h⁻¹ MD |
| Mehler et al., 2024 | Chile | RCT | dNCR | DEX vs no DEX | 74.5[71.0,83.0] vs 82.0[70.0,93.0] | 54.0% vs 20.0% | 23 (13 vs 10) | Non-neurosurgical and non-cardiac surgeries (orthopedic, vascular, abdominal, urogenital, gynecological surgeries) | GA | 0.8 μg・kg⁻¹・h⁻¹ MD |
| Li et al., 2024 | China | Retrospective Cohort | dNCR | DEX + ropivacaine vs ropivacaine | 71.3±7.2 vs 70.8±6.9 | 48.2% vs 57.1% | 112(56 vs 56) | Elective THA procedure | Pericapsular nerve group block (PNGB) + GA | 1 μg/kg BA |
| Huo et al., 2024 | China | Retrospective Cohort | dNCR | DEX vs DEX+ulinastatin vs ulinastatin | 71.7±2.5 vs 72.3±2.7 vs 71.2±4.9 | 48.0% vs 45.0% vs 49.0% | 397 (129 vs 118 vs150) | Unilateral THA | GA | 0.3 μg・kg⁻¹・h⁻¹ MD |
| Bu et al., 2024 | China | RCT | dNCR | DEX vs saline | 71.4±4.3 vs 70.8±4.5 | 66.1% vs 54.2% | 117(59 vs 58) | Elective radical resection of colon cancer | GA | 1 μg/kg LD then 0.3 μg・kg⁻¹・h⁻¹ MD |
| Wu et al., 2023 | China | RCT | POD; dNCR | DEX vs saline | 69.0[67.0,74.0] vs 70.0[67.0,73.0] | 40.0% vs 34.5% | 110(55 vs 55) | Total hip/knee arthroplasty | GA + Lumbar plexus-sciatic nerve block | 2.0 μg/kg intranasal |
| Wei and Guo, 2023 | China | Retrospective Cohort | POD; dNCR | low-dose DEX vs medium-dose DEX vs high-dose DEX | 68.4±2.2 vs 67.4±2.8 vs 67.9±2.3 | 54.0% vs 56.0% vs 53.0% | 112(35 vs 39 vs 38) | Lower extremity orthopedic surgery | Combined spinal-epidural anesthesia (CSEA) | 0.2 μg/kg, 0.4μg/kg, 0.6μg/kg LD |
| Liao et al., 2023 | China | RCT | dNCR | DEX vs saline vs remimazolam | 71.3±3.6 vs 69.7±2.5 vs 70.1±3.6 | 57.1% vs 60.0% vs 61.8% | 104(35 vs 35 vs 34) | Laparoscopic radical resection of gastric cancer | GA + Bilateral transverse abdominal plane blocks (BTAP) + Rectus abdominis sheath blocks (RASB) | 0.5 μg/kg LD then 0.3-0.5 μg・kg⁻¹・h⁻¹ MD |
| Kurup et al., 2023 | India | RCT | dNCR | DEX vs lidocaine | 65.0[61.0,72.0] vs 64.0[60.0,70.0] | 27.6% vs 35.5% | 60(29 vs 31) | Open abdominal surgery | GA + Epidural anesthesia (EA) | 0.5 μg/kg LD then 0.5 μg・kg⁻¹・h⁻¹ MD |
| Gao et al., 2022 | China | RCT | dNCR; pNCD | DEX vs saline | 68.2±6.0 vs 67.2±5.2 | 13.0% vs 19.0% | 95(47 vs 48) | Unilateral TKA | GA + Femoral Nerve Block (FNB) | 1 μg/kg LD then 0.4 μg・kg⁻¹・h⁻¹ MD; 2 µg/kg PCIA |
| Xin et al., 2021 | China | RCT | POD | DEX vs saline | 69.0[67.0,70.0] vs 68.0[66.0,71.0] | 30.0% vs 43.3% | 60(30 vs 30) | Laparoscopic cholecystectomy | GA | 0.5 μg/kg LD then 0.4 μg・kg⁻¹・h⁻¹ MD |
| Lu et al., 2021 | China | RCT | POD | DEX vs saline | 70.1±5.8 vs 70.4±6.5 | 64.5% vs 67.4% | 675(344 vs 331) | Elective abdominal surgery (gastric surgery, intestinal surgery, hepatobiliary surgery, pancreatic surgery, or appendectomy) | GA | 0.5 μg/kg LD then 0.2 μg・kg⁻¹・h⁻¹ MD |
| Hu et al., 2022 | China | RCT | POD | DEX + TIVA vs TIVA | 69.6±4.5 vs 69.1±5.1 | 83.3% vs 81.6% | 177(90 vs 87) | Transthoracic oesophagectomy | GA | 0.4 μg/kg LD then 0.1 μg・kg⁻¹・h⁻¹ MD |
| Yin et al., 2020 | China | Retrospective Cohort | POD | DEX vs saline | 72.2±0.3 vs 72.1±0.2 | 58.1% vs 55.2% | 120(62 vs 58) | Hip fracture surgery | GA | 0.5 μg/kg LD then 0.4 μg・kg⁻¹・h⁻¹ MD |
| Wang et al., 2020 | China | RCT | dNCR | DEX vs saline | 68.4±3.3 vs 68.3±2.1 | 63.3% vs 60.0% | 110(60 vs 50) | Radical gastrectomy | GA | 0.5 μg/kg LD then 0.4 μg・kg⁻¹・h⁻¹ MD |
| Li et al., 2020 | China | RCT | dNCR | DEX vs no DEX | 67.4±3.3 vs 67.3±2.1 | 51.2% vs 52.2% | 87(41 vs 46) | Lung cancer resection | GA | 0.5 μg/kg LD then 0.1 μg・kg⁻¹・h⁻¹ MD |
| Li Bao, 2020 | China | RCT | dNCR | DEX vs DEX + ulinastatin | 64.9±5.7 vs 65.3±5.7 | 57.8% vs 54.7% | 178(83 vs 95) | Laparoscopic colorectal cancer surgery | GA | 0.5 μg/kg LD then 0.3 μg・kg⁻¹・h⁻¹ MD |
| Wang et al., 2019 | China | RCT | dNCR; pNCD | DEX vs Midazolam | 70.5±5.0 vs 69.4±4.5 | 67.0% vs 66.0% | 198(98 vs 100) | Selective orthopedic surgery, abdominal surgery, and thoracic surgery | GA | 0.5 μg/kg LD |
| Mansouri et al., 2019 | Iran | RCT | dNCR | DEX vs midazolam vs saline | 66.5±1.6 vs 63.6±8.3 vs 64.0±7.3 | 50.0% vs 50.0% vs 38.0% | 150(50 vs 50 vs 50) | Cataract surgery | GA | 1 μg/kg IV |
| Mei et al., 2018 | China | RCT | POD; dNCR | DEX vs propofol | 76.0±7.0 vs 74.0±6.0 | 48.0% vs 43.2% | 296(148 vs 148) | Total hip arthroplasty | GA + Peripheral nerve block (PNB) | 0.8-1.0 μg/kg LD then0.1-0.5 μg・kg⁻¹・h⁻¹ MD |
| Deiner et al., 2017 | America | RCT | POD; pNCD | DEX vs saline | 74.0[71.0,78.0] vs 74.0[71.0,78.0] | 48.7% vs 48.8% | 390(189 vs 201) | Major elective noncardiac surgery (spine, thoracic, orthopedic, urologic, general surgery) | GA | 0.5 μg・kg⁻¹・h⁻¹ MD |
| Chen et al., 2015 | China | Retrospective Cohort | dNCR | DEX vs saline | 70.6±4.2 vs 71.4±4.9 | 64.4% vs 62.3% | 148(87 vs 61) | Elective noncardiac surgery (including fracture surgery, prostate removal, gallbladder surgery, radical resection of rectal carcinoma) | GA | 0.5 μg/kg LD then 0.2 μg・kg⁻¹・h⁻¹ MD |
| Chen et al., 2013 | China | RCT | dNCR; pNCD | DEX vs saline | 66.2±7.5 vs 67.9±6.6 | 55.9% vs 49.2% | 122(59 vs 63) | Laparoscopic cholecystectomy | GA | 1 μg/kg LD then 0.4 μg・kg⁻¹・h⁻¹ MD |

**Notes:** BA, block administration; DEX, Dexmedetomidine; dNCR, Delayed neurocognitive recovery; ERCP, endoscopic retrograde cholangiopancreatography; GA, general anesthesia; IV, intravenous; LD, Loading dose; MD, Maintenance dose; PCIA, patient-controlled intravenous analgesia; pNCD, Postoperative neurocognitive disorders; PND, Perioperative neurocognitive disorders; POD, Postoperative delirium; RCT, randomized controlled trial; THA, Total hip arthroplasty; TKA, Total knee arthroplasty; TIVA, total intravenous anaesthesia. Note: The age data in this table are presented in the format of each study's original report, including ‘(Mean ± SD) (x̅ ± s)’ or ‘[Median (IQR)] [M(P25,P75)]’

# References

Bu, H.-M., Zhao, M., Ma, H.-M., and Tian, X.-P. (2024). Application value of dexmedetomidine in anesthesia for elderly patients undergoing radical colon cancer surgery. *World J. Gastrointest. Surg.* 16, 2671–2678. doi: 10.4240/wjgs.v16.i8.2671

Chen, J., Yan, J., and Han, X. (2013). Dexmedetomidine may benefit cognitive function after laparoscopic cholecystectomy in elderly patients. *Exp. Ther. Med.* 5, 489–494. doi: 10.3892/etm.2012.811

Chen, W., Liu, B., Zhang, F., Xue, P., Cui, R., and Lei, W. (2015). The effects of dexmedetomidine on post-operative cognitive dysfunction and inflammatory factors in senile patients. *Int. J. Clin. Exp. Med.* 8, 4601–4605.

Deiner, S., Luo, X., Lin, H.-M., Sessler, D. I., Saager, L., Sieber, F. E., et al. (2017). Intraoperative infusion of dexmedetomidine for prevention of postoperative delirium and cognitive dysfunction in elderly patients undergoing major elective noncardiac surgery: a randomized clinical trial. *JAMA Surg.* 152, e171505. doi: 10.1001/jamasurg.2017.1505

Evered, L., Silbert, B., Knopman, D. S., Scott, D. A., DeKosky, S. T., Rasmussen, L. S., et al. (2018). Recommendations for the nomenclature of cognitive change associated with anaesthesia and surgery-2018. *Br. J. Anaesth.* 121, 1005–1012. doi: 10.1016/j.bja.2017.11.087

Gao, C., Huang, T., Wu, K., Zhang, W., Wang, S., Chai, X., et al. (2022). Multimodal analgesia for accelerated rehabilitation after total knee arthroplasty: a randomized, double-blind, controlled trial on the effect of the Co-application of local infiltration analgesia and femoral nerve block combined with dexmedetomidine. *Brain Sci.* 12, 1652. doi: 10.3390/brainsci12121652

Hao, X., Zhang, Z., Yang, L., Guo, Y., Cao, F., Cao, J., et al. (2025). Association of dexmedetomidine with postoperative depressive symptoms in older surgical patients: a prospective multicenter study. *CNS Neurosci. Ther.* 31, e70407. doi: 10.1111/cns.70407

Hu, J., Zhang, Y., and Maze, M. (2022). Dexmedetomidine for prevention of postoperative delirium in older adults undergoing oesophagectomy with total intravenous anaesthesia. *Eur. J. Anaesthesiol.* 39, 296. doi: 10.1097/EJA.0000000000001648

Huo, Q.-F., Zhu, L.-J., Guo, J.-W., Jiang, Y.-A., and Zhao, J. (2024). Effects of ulinastatin combined with dexmedetomidine on cognitive dysfunction and emergence agitation in elderly patients who underwent total hip arthroplasty. *World J. Psychiatry* 14, 26–35. doi: 10.5498/wjp.v14.i1.26

Kurup, M. T., Sarkar, S., Verma, R., Bhatia, R., Khanna, P., Maitra, S., et al. (2023). Comparative evaluation of intraoperative dexmedetomidine versus lidocaine for reducing postoperative cognitive decline in the elderly: a prospective randomized controlled trial. *Anaesthesiol. Intensive Ther.* 55, 349–357. doi: 10.5114/ait.2023.134251

Li Bao, K. G., Xiaowei Tian, Jing Zhang, Li Chen (2020). Effects of Ulinastatin Combined with Dexmedetomidine on Postoperative Cognitive Function and Central Nerve Specific Protein Level in Elderly Colorectal Cancer Patients after Laparoscopic. *Oncologie* 22, 167–178. doi: 10.32604/oncologie.2020.012495

Li, X., Chen, L., Sun, Y., and Li, Y. (2024). Effects of dexmedetomidine added to ropivacaine in ultrasound-guided continuous pericapsular nerve group block among elderly patients undergoing total hip arthroplasty. *Rejuvenation Res.* 27, 115–121. doi: 10.1089/rej.2024.0014

Li, Y., Wang, C., Bi, M., Gao, J., Zhang, X., and Tian, H. (2020). Effect of dexmedetomidine on brain function and hemodynamics in patients undergoing lung cancer resection. *Oncol. Lett.* 20, 1077–1082. doi: 10.3892/ol.2020.11675

Liao, Y. Q., Min, J., Wu, Z. X., and Hu, Z. (2023). Comparison of the effects of remimazolam and dexmedetomidine on early postoperative cognitive function in elderly patients with gastric cancer. *Front. Aging Neurosci.* 15, 1123089. doi: 10.3389/fnagi.2023.1123089

Lu, Y., Fang, P.-P., Yu, Y.-Q., Cheng, X.-Q., Feng, X.-M., Wong, G. T. C., et al. (2021). Effect of Intraoperative Dexmedetomidine on Recovery of Gastrointestinal Function After Abdominal Surgery in Older Adults: A Randomized Clinical Trial. *JAMA Netw Open* 4, e2128886. doi: 10.1001/jamanetworkopen.2021.28886

Mansouri, N., Nasrollahi, K., and Shetabi, H. (2019). Prevention of cognitive dysfunction after cataract surgery with intravenous administration of midazolam and dexmedetomidine in elderly patients undergoing cataract surgery. *Adv. Biomed. Res.* 8, 6. doi: 10.4103/abr.abr_190_18

Mehler, D. M., Kreuzer, M., Obert, D. P., Cardenas, L. F., Barra, I., Zurita, F., et al. (2024). Electroencephalographic guided propofol-remifentanil TCI anesthesia with and without dexmedetomidine in a geriatric population: electroencephalographic signatures and clinical evaluation. *J. Clin. Monit. Comput.* 38, 803–815. doi: 10.1007/s10877-024-01127-4

Mei, B., Meng, G., Xu, G., Cheng, X., Chen, S., Zhang, Y., et al. (2018). Intraoperative sedation with dexmedetomidine is superior to propofol for elderly patients undergoing hip arthroplasty: a prospective randomized controlled study. *Clin. J. Pain* 34, 811–817. doi: 10.1097/AJP.0000000000000605

Sun, Z., Shi, J., Liu, C., Zhang, J., Liu, Y., Wu, Y., et al. (2024). The effect of low-dose dexmedetomidine on perioperative neurocognitive dysfunction in elderly patients undergoing endoscopic retrograde cholangiopancreatography (ERCP): a randomized, controlled, double-blind trial. *Drug Des. Dev. Ther.* 18, 3715–3725. doi: 10.2147/DDDT.S470514

Tao, Q.-Y., Liu, D., Wang, S.-J., Wang, X., Ouyang, R.-N., Niu, J.-Y., et al. (2024). Effects of esketamine combined with dexmedetomidine on early postoperative cognitive function in elderly patients undergoing lumbar spinal surgery: a double-blind randomized controlled clinical trial. *Drug Des. Dev. Ther.* 18, 5461–5472. doi: 10.2147/DDDT.S481173

Wang, W., Feng, N., Zhao, W., Luo, F., Zhu, X., Zhao, W., et al. (2019). Dexmedetomidine reduces brain neuronal injuries but not clinical neurocognitive function in the elderly , compared to midazolam. Available at: https://api.semanticscholar.org/CorpusID:199367420

Wang, Z., Shen, Z., Wang, H., Zhang, L., and Dong, R. (2020). Effect of dexmedetomidine on the cognitive function of patients undergoing gastric cancer surgery by regulating the PI3K/AKT signaling pathway. *Oncol. Lett.* 19, 1151–1156. doi: 10.3892/ol.2019.11224

Wei, W., and Guo, L. (2023). Effect of dexmedetomidine on convalescence quality after general anesthesia and postoperative delirium, and on cognitive function in elderly patients undergoing lower limb surgery. *Tropical Journal of Pharmaceutical Research* 22, 2193–2200. doi: 10.4314/tjpr.v22i10.22

Wu, J., Liu, X., Ye, C., Hu, J., Ma, D., and Wang, E. (2023). Intranasal dexmedetomidine improves postoperative sleep quality in older patients with chronic insomnia: a randomized double-blind controlled trial. *Front. Pharmacol.* 14, 1223746. doi: 10.3389/fphar.2023.1223746

Xin, X., Chen, J., Hua, W., and Wang, H. (2021). Intraoperative dexmedetomidine for prevention of postoperative delirium in elderly patients with mild cognitive impairment. *Int. J. Geriatr. Psychiatry* 36, 143–151. doi: 10.1002/gps.5406

Ye, Q., Hu, Y., Xing, Q., Wu, Y., and Zhang, Y. (2024). The effects of opioid-free anesthesia with dexmedetomidine and esketamine on postoperative anesthetic-related complications for hip surgery in the elderly. *Int. J. Gen. Med.* 17, 6291–6302. doi: 10.2147/IJGM.S492771

Yin, L., Yuan, H., Chen, X., Liao, M., and Lu, F. (2020). Mechanism of action of dexmedetomidine on hemodynamics, analgesic and sedative effects and postoperative delirium in elderly patients undergoing hip fracture surgery. Available at: https://api.semanticscholar.org/CorpusID:226951509

Zhang, X., Zhang, J., Gu, W., Wu, D., Shi, C., and Ma, Z. (2024). Dexmedetomidine adjunct to ropivacaine for ultrasound-guided transversus abdominis plane block for open inguinal hernia repair in the older adults: a randomised clinical trial. *J. Minimal Access Surg.* 20, 187–195. doi: 10.4103/jmas.jmas_189_22
